# Polyphenolic Characterization of Nebbiolo Red Wines and Their Interaction with Salivary Proteins

**DOI:** 10.3390/foods9121867

**Published:** 2020-12-15

**Authors:** Joana Azevedo, Elsa Brandão, Susana Soares, Joana Oliveira, Paulo Lopes, Nuno Mateus, Victor de Freitas

**Affiliations:** 1 LAQV/REQUIMTE—Laboratório Associado para a Química Verde, Faculdade de Ciências da Universidade do Porto, Rua do Campo Alegre 687, 4169-007 Porto, Portugal; up200300957@fc.up.pt (J.A.); elsa.brandao@fc.up.pt (E.B.); susana.soares@fc.up.pt (S.S.); jsoliveira@fc.up.pt (J.O.); nbmateus@fc.up.pt (N.M.); 2 Amorim Cork S.A., Rua dos Corticeiros 830, 4536-904 Santa Maria de Lamas, Portugal; paulo.lopes@amorim.com

**Keywords:** wine, tannins, anthocyanins, proline-rich proteins

## Abstract

The present study correlates the polyphenolic composition of two different Nebbiolo red wines from the 2015 vintage (M and P), with the salivary proteins’ precipitation process. The work centered on the polyphenolic characterization of Nebbiolo wines and their interaction with different families of salivary proteins. Overall, both wines were found to be very reactive with human saliva which was supposed to contribute to their astringent character. The comparison of both wines showed that the M wine presented higher values of total phenolics, total proanthocyanidins, and tannin specific activity. Moreover, this wine showed a higher interaction with salivary proteins. Altogether, the chemical characterization and reactivity toward human saliva could contribute to the wine astringency.

## 1. Introduction

Polyphenols are the main compounds present in grapes and wines which are responsible for some of their organoleptic properties such as color, bitterness, and astringency [1,2,3]. Within these compounds, phenolic acids and derivatives, anthocyanins, and flavan-3-ols (catechin monomers and proanthocyanidins) are the main compounds present in red grapes [4].

The major contributors of red color in wines are anthocyanins or their further derivatives which are extracted or formed during the vinification process [5,6]. It is known that the reaction between anthocyanins and proanthocyanidins occurs mainly during conservation and aging. The pigments formed during this period are more stable than their anthocyanin precursors and have different sensory properties [7,8,9,10].

Red wines also contain high amounts of tannins (condensed tannins also known as proanthocyanidins) and their content in wines arises mainly from their extraction from grapes during the winemaking process. In addition, tannins such as hydrolysable tannins, can also be extracted from oak wood during wine aging in oak barrels [11,12] or from the use of oenological additives. Moreover, due to their ability to bind proteins [12], tannins can directly affect the wine astringency [3,12,13,14]. Astringency is defined as dry, rough, pucker, or grippy and is a tactile sensation that usually persists in the mouth; its intensity may increase depending on the saliva flow rate [3,15,16,17]. The constitutive characteristics of tannins and their concentration influence red wine astringency perception. A high mean degree of polymerization or a large amount of galloyl groups in the tannin structure, and a high tannin concentration induce a higher intensity of astringency and dryness perception [18], whereas pigmented tannins seem to reduce it [19,20,21]. The astringency descriptors induced by ellagitannins have been reported to be different from the ones yielded by condensed tannins [22], and have been described as a rather mellow [14,23] and smooth astringency with a velvety mouth-coating sensation [13], with an impact in the roundness and amplitude of red wines [3,24,25]. One of the key mechanisms for astringency perception is the interaction and precipitation of salivary proteins, in particular the proline-rich proteins (PRPs). This family of salivary proteins is characterized by a high content in proline residues and are classically divided into basic PRPs (bPRPs), acidic PRPs (aPRPs), and glycosylated PRPs (gPRPs). Although bPRPs are usually linked to astringency perception, some other studies have shown that aPRPs were significantly precipitated by tannins [22]. In addition to aPRPs, mucins, statherin, and P-B peptide have also been shown to have a significant interaction with tannins.

The present study aims to deepen the phenolic compound composition of two Nebbiolo red wines (P and M) from the 2015 vintage and to understand the complexation process with salivary proteins. It is intended to compare these wines in terms of some physical-chemical parameters commonly related to the astringency perception in order to differentiate them (tannins concentration and salivary protein complexation).

## 2. Materials and Methods

### 2.1. Reagents

The L-(+)-tartaric acid (99%), ethyl acetate (99.9%), methanol (99.8%), acetonitrile (99.8%), acetic acid (99.7%), Folin–Ciocalteu reagent, and sodium bisulfite were obtained from Sigma-Aldrich, Madrid, Spain. Ethanol was purchased from AGA^®^ (96%), Prior Velho, Portugal and HCl 37% from Fluka^®^, College Park, MD, USA.

### 2.2. Wine Samples

The commercial wine coded as M was produced during the 2015 vintage from 100% Nebbiolo grapes sourced from La Morra and Castiglione Falletto, Barolo, Piemont (Italy). This wine underwent skins maceration in stainless steel tanks at a controlled temperature for 12 days before pressing and alcoholic and malolactic fermentation for around 20 days.

The wine was aged in small barrels of oak for 24 months. After decanting, the wine was bottled without fining or filtration. The commercial wine coded as P was produced during the 2015 vintage from 100% Nebbiolo grapes sourced predominantly from the Comune di Serralunga d’Alba, Piemont (Italy). For vinification, the pressed grapes fermented without yeast inoculation and without sulfur for about a month. The wines were left to age for twenty-four months, in barrels of 40 hectoliters. The bottles of each wine were sent directly from producers and opened in 2019 (4 years old). Both wines were considered to be very astringent by an expert panel of wine tasters and it was not possible to distinguish them in terms of that sensation.

### 2.3. Total Phenolic Compounds

Total phenolic compounds was determined using the Folin–Ciocalteu assay, according to the method described by [26] and adapted from [27]. In a microtube, 610 μL of distilled water, 15 μL of wine, and 75 μL of Folin–Ciocalteu reagent were mixed. After 30 s, 300 μL of aqueous 20% Na_2_CO_3_ and 300 μL of distilled water were added, and the mixture was mixed (30 s) and allowed to stand at room temperature in the dark for 30 min. The absorbance at 750 nm was determined, and the total phenolic compounds concentration was calculated accordingly to the calibration curve as follows: Abs_blank_ (750 nm) − Abs_sample_ (750 nm) = 0.9638 × concentration (mM) − 0.0001, R^2^ = 0.9999, using gallic acid as standard.

### 2.4. Total Proanthocyanidins

Total proanthocyanidins (condensed tannins) was determined based on the Bate-Smith reaction, slightly adapted as described previously [28]. This method consisted of the measurements of sample absorbance (at 520 nm) of anthocyanidins resulting from the acidic decomposition of condensed tannins, at 100 °C for 30 min, in strongly acidic conditions [29].

Unknown concentrations of condensed tannins in wines were determined using a calibration curve (R^2^ = 0.9911) obtained using different concentrations (ranging from 0.264 to 5.92 mg/mL) of a procyanindin fraction composed of dimeric and trimeric procyanidins purified from grape seeds [28]. Each sample was prepared in duplicate and injected in triplicate and results were expressed as mean ± standard deviation and presented as g·L^−1^ equivalent of total procyanidins.

### 2.5. Identification and Quantification of Phenolic Compounds by High Performance Liquid Cromatography (HPLC)-Diode Array Detector (DAD) and Liquid Chromatography-DAD/Electron Spray Ionization (ESI)-Mass Spectrometry (MS) Analysis

For the phenolic and cinnamic acids and their derivatives identification and quantification, red wine samples were extracted by micro liquid-liquid extraction, performed according to the procedure described by [30]. For that, 600 µL of wine, 600 µL of ethyl acetate, and 300 µL of acetonitrile were added to a microtube and vortexed for 10 s, and then the samples were centrifuged for 5 min at 5400× *g*. Organic and aqueous phases were separated, and then the same procedure was performed for the remaining aqueous phase. Both organic fractions were combined, and the organic solvent was removed using a CentriVac Concentrator Labconco, Kansas City, MO, USA system and resuspended in 50:50 (*v*/*v*) methanol/water. Each wine extraction was performed in duplicate. The method used had been previously validated [30].

Samples were analyzed by HPLC-DAD, following the method described in the literature [31], using a HPLC (Merck^®^ Hitachi Elite Lachrom, Kenilworth, NJ, USA) on a 150 × 4.6 mm i.d. reversed-phase C18 column (Merck^®^) thermostated at 25 °C (Merck^®^ Hitachi Column Oven L-2300). Detection was carried out at 280 nm using a diode array detector (Merck^®^ Hitachi Diode Array Detector L-2455). Solvents were (A) H_2_O/CH_3_COOH (99:1) and (B) CH_3_COOH/CH_3_CN/H_2_O (1:20:79) with the gradient 80–20% A over 55 min, 20–10% A from 55 to 70 min, and 10–0% A from 70 to 90 min, at a flow rate of 0.3 mL/min. The sample injection volume was 20 μL. The chromatographic column was washed with 100% B for 10 min, and then stabilized with the initial conditions for another 10 min.

The concentration of each compound in the different wine samples was obtained as equivalents of gallic acid using the following calibration curve: [Phenolic Compound]_mM_ = (peak area + 0.0831)/329.09, R^2^ = 0.9999. This curve was obtained using commercial gallic acid in the range 0.005–1 mM. The analysis was made in triplicate.

Anthocyanins and anthocyanin-derived pigments were quantified by HPLC-DAD, using the same HPLC equipment described previously. In this case, we used a 250 × 4.6 mm i.d. reversed-phase C18 column (Merck^®^), and the following solvents were used: (A) 7.5% (*v*/*v*) formic acid in water and (B) 7.5% (*v*/*v*) formic acid in acetonitrile. The gradient consisted of 97–70% A for 31 min, the column was washed with 100% solvent B for 10 min, and equilibrated with the initial conditions for another 10 min. The flow rate was 1 mL min^−1^ and detection was carried out at 520 and 511 nm. For the quantification of anthocyanins, a calibration curve using different concentrations of malvidin-3-*O*-glucoside (16.5–1059.9 mg/L) was used, i.e., [malvidin-3-*O*-glucoside]_mM_ = (peak area + 874484)/18687 R^2^ = 0.9998. For the quantification of anthocyanin-derived pigments, A-type vitisin (carboxypyranomalvidin-3-*O*-glucoside) was used as a standard (2.5–25 mg/L). [Carboxypyranomalvidin-3-*O*-glucoside]_mM_ = (peak area + 19302)/47027 R^2^ = 0.996.

The identification of the compounds in all samples was performed by LC-DAD/ESI-MS using the same solvents, gradients, injection volume, and flow rate referred to above for the identification and quantification of phenolic compounds by HPLC analysis. Double-online detection was done by a photodiode spectrophotometer and mass spectrometry, as described by some of us [31].

### 2.6. Color Index (Red Color Index (RCI) and CIELab)

The color characteristics were evaluated by the red color index (RCI) at A520 nm and CIELab color coordinates (L*, C*, and H*), where L* is the lightness, C* the chromaticity, and H* hue, measured by direct absorption and determined by Color Win-MSCV^®^ Coordinates software, with a 2 mm optical path read absorbance at 450, 520, 570, and 630 nm [32].

### 2.7. Tannin Specific Activity (TSA)

Tannin specific activity (TSA) determines the phenolic compounds capacity to precipitate a protein (bovine serum albumin (BSA)) measured by nephelometry, as described elsewhere [33]. Red wines were diluted 1:50 with a wine model solution (12% ethanol, 5.0 g L^−1^ tartaric acid, pH 3.20), and filtered (0.45 µm). Then, 4.0 mL of this solution were transferred to a turbidimetry tube and the nephelometry values were recorded in Turbidimeter HACH 2100 N, Lisbon, Portugal adapted for cells of 100 × 12 mm. Then, 150 µL of BSA (0.8 mg/mL) was added to each tube and vortexed. The tubes were kept in the dark for 30 min, and then the maximum turbidity was determined. The TSA was expressed in turbidity units NTU/mL of wine and was determined by the following expression, where 0.08 corresponds to the dilution factor of wine: Turbidity (NTU/mL of wine) = (turbidity after BSA − turbidity t0)/0.08.

### 2.8. Interaction of Wine with Human Saliva

Human saliva was collected and treated, as reported elsewhere [22]. Briefly, fifteen healthy subjects were instructed to avoid food or beverages intake at least 1 h prior to saliva isolation. Saliva was collected at 2:00 p.m., pooled together, and treated with Trifluoracetic acid (TFA) (final concentration 0.1%) to precipitate high molecular weight proteins (e.g., mucin and amylase) and to inhibit proteases. After mixing, saliva was centrifuged (8000× *g*, 5 min) and the supernatant, referred to as acidic saliva (AS), was recovered. The total protein concentration was assessed by the Bradford assay using BSA as the standard and was determined to be 584 µg/mL. Then, this AS was used for the interactions with the wine samples. The interaction with P and M wines was made using a fixed volume of AS (90.0 µL) to maintain the concentration of salivary proteins (438 µg/mL). Wine model solution (WMS) or wine (30.0 µL) were added to 90.0 µL of AS. The wines were added at different ratios (saliva/wine, *v*/*v*: 10:0.7, 10:2, and 10:3) and the necessary volume of WMS was added to attain a final volume of 30.0 µL. The solutions were mixed and reacted for 5 min at room temperature. Then, the solutions were centrifuged (8000× *g*, 5 min) and the supernatant was analyzed. The following six families of SP were monitored using HPLC-Ultra-Violet (UV): bPRPs, gPRPs, aPRPs, cystatins, statherin, and P-B peptide. As statherin and P-B peptide were co-eluted, they were analyzed together. One hundred microliters of each supernatant were analyzed on a HPLC Lachrom system (Merck^®^ Hitachi, L-7100) equipped with Kinesis C8 column (150 × 2.1 mm, 5 µm particle diameter) and a UV-Visivel detector (L-7420). The HPLC conditions were as follows: eluent (A) 0.2% aqueous TFA, eluent (B) 0.2% TFA in acetonitrile/water 80:20 (*v*/*v*); linear gradient was 10 to 45% of B in 40 min, followed by a washing step for 10 min with 100% eluent B and stabilization on the initial conditions; flow rate of 0.50 mL/min; detection at 214 nm.

Preceding the human saliva isolation, an informed consent was given to all the participants to sign. Consent forms were obtained for all participants. The study was approved by the Ethics Committee of the University of Porto (CES183/18) and conducted according to the Declaration of Helsinki.

### 2.9. Statistical Analysis

All determinations were conducted in triplicate. Values are expressed as the arithmetic means ± standard deviation. Statistical significance of the difference between the two wines was evaluated by *t*-test with Welch’s correction or ANOVA (followed by Bonferroni) using the GraphPad Prism v7.0. Differences were considered to be significant when *p* < 0.05.

## 3. Results

The main goal of this work was to explore the phenolic compound composition of two Nebbiolo red wines from the 2015 vintage and to understand their involvement in the complexation process with salivary proteins. For that, the phenolic compositions of M and P wines were firstly determined, concerning the total phenolic compounds, the total condensed tannins, and the concentration of anthocyanins and anthocyanin-derived pigments. Then, this phenolic composition was related to the wines’ reactivity towards different families of salivary proteins, as is further discussed.

### 3.1. Phenolic Composition of Red Nebbiolo Wines

The most abundant phenolic compounds that are described to be present in wines are flavanols, anthocyanins, and phenolic acids [34,35,36]. Anthocyanins are responsible for the color of red wines, especially young red wines and, during aging, the concentration of these pigments decreases, yielding to the formation of anthocyanin derivatives and to a color change from a red-violet hue to a more red-orange hue.

The chromatic features of M and P wines were evaluated using the CIELab system that uses Cartesian coordinates to calculate color in a color space, determining the lightness (L*) and the chromaticity (C*) composed of the following two parameters: a* (green for red) and b* (blue for yellow) and H° represents the hue. Table 1 shows the CIELab parameters obtained for both wines and it can be observed that M wine presents lower luminosity (L*) and higher chromaticity (C*) values as compared with P wines. Although both wines present orange hues, M wines presented higher values of a* and b* coordinates which was indicative of a more orange hue than P wines and this was associated with the wines’ age (4-years old).

**Figure 1:**
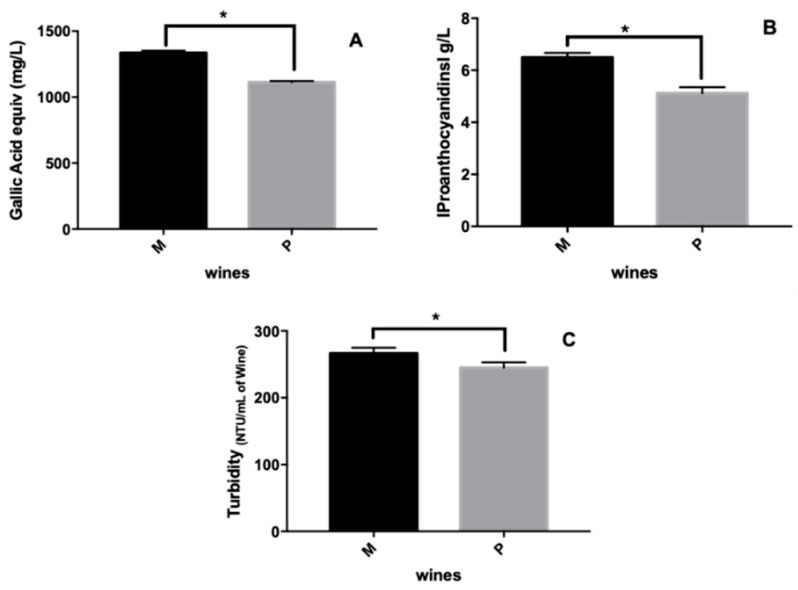
Graphical representation of the mean quantification ± standard deviation. (**A**) Total phenolic compounds (determined by the Folin–Ciocalteu method); (**B**) Total proanthocyanidins (determined using the Bath-Smith method); (**C**) Tannin specific activity for M and P wines samples. The presented values are the mean of three independent experiments, * Represents statistically different (*p* < 0.0001). M and P refer to the wine codes.

Moreover, the anthocyanin concentrations of M and P wines were determined by HPLC-DAD and, as observed in Table 1, the concentration of anthocyanins in M wines was significantly lower than in P wines. These results are not correlated with the absorbance at 520 nm (Table 1) observed for both wines, as M wines present a higher value (0.581) as compared with P wines (0.373). This behavior can be related to the presence of more complex anthocyanin structures in M wines not detected by HPLC. It is well known that, during the wine aging process, the concentration of anthocyanins decreases yielding to the formation of anthocyanin-derived pigments such as A- and B-type vitisin and tannins-anthocyanin condensation products, as already reported in the literature [7,8,9], that are responsible for wine color stabilization. In fact, Table 1 shows that along with the lowest concentration of anthocyanins observed in M wines, it is possible to detect a greater amount of A-type vitisins, probably resulting from a higher color evolution as compared with P wine. Moreover, the higher amounts of polyphenols, and in particular, of condensed tannins present in M wines (Figure 1) that can act as copigment and yield to anthocyanin color stabilization by copigmentation, may also explain the higher absorbance at 520 nm. Moreover, both wines are rich in benzoic and cinnamic acids and some derivatives that are also described to be good copigments toward red color stabilization of anthocyanins (Table 2) [21].

Regarding the ability of wines to precipitate BSA (Figure 1C), although both wines yielded BSA precipitation, M wine showed a higher ability to precipitate BSA. Indeed, the higher level of polyphenols such as anthocyanins, phenolic acids, and in particular, condensed tannins, can explain this behavior. This higher TSA suggests that M wine could present a more astringent character.

### 3.2. Interaction of Wines with Salivary Proteins

**Figure 2:**
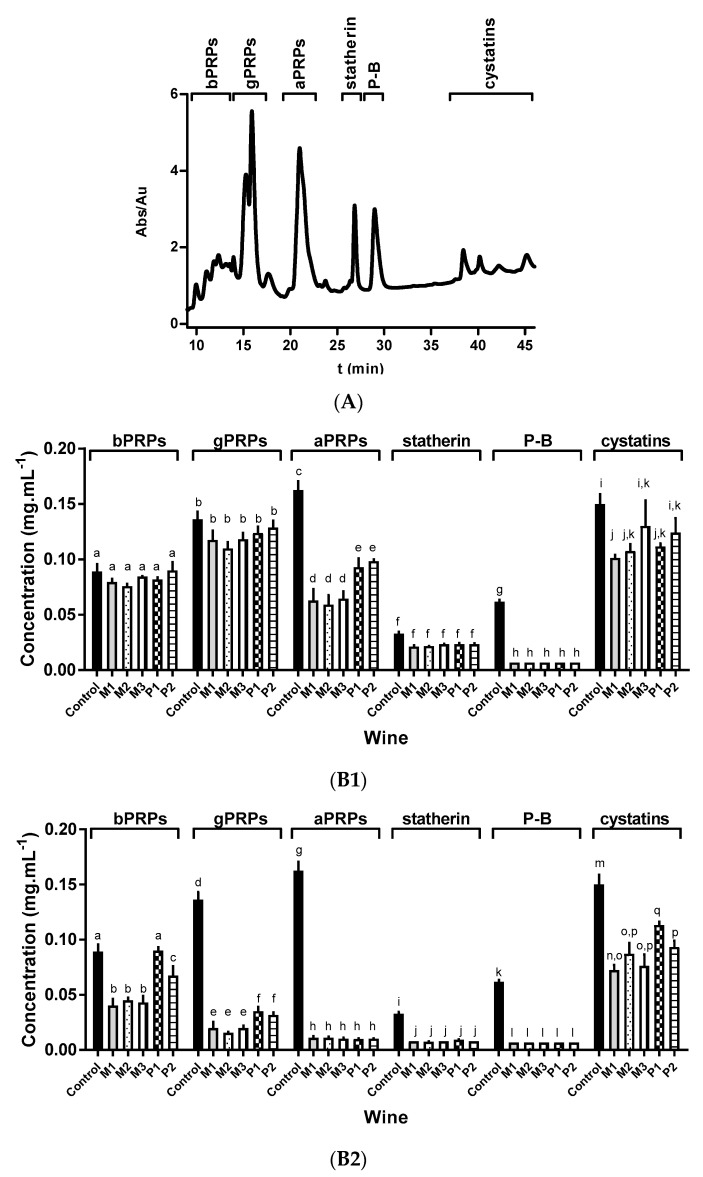

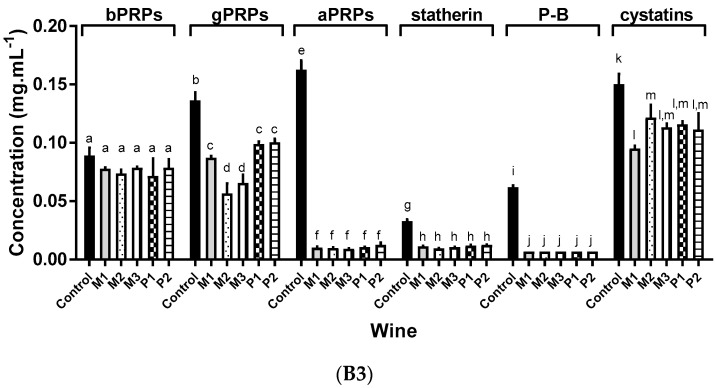
(**A**) Typical salivary protein profile obtained by HPLC analysis with detection at 214 nm, and identification of the major families eluted along the chromatogram, the identification of the different families has been previously reported [22,37]; bPRPs: basic proline-rich proteins, gPRPs: glycosylated proline-rich proteins, aPRPs: acidic proline-rich proteins. (**B**) Modifications in the concentration (mg mL^−1^) for each family of salivary proteins upon the interaction with each wine (two bottles of M wine (M1 and M2) and three bottles of P wine (P1, P2, and P3)) at different ratios (**B1**) 10:0.7, (**B2**) 10:2, and (**B3**) 10:3. Control condition is the concentration of each family of salivary proteins in human saliva in the absence of wine. Data are presented as mean and SEM of at least three independent experiments (values with the same letter are not significantly different, *p* < 0.05, ANOVA with Bonferroni correction for multiple comparisons).

To better understand the chemical differences of M and P wines and to differentiate them in terms of their ability to interact with salivary proteins, studies involving interactions between M and P wines with human saliva were performed. Different saliva/wine ratios (10:0.7, 10:2, and 10:3) were mixed and left to interact for 5 min. Then, a centrifugation was carried out to remove the eventually formed insoluble complexes and the soluble salivary proteins that remained in the supernatant were analyzed by HPLC-UV (Figure 2).

From Figure 2, it is possible to observe that the addition of different volumes of each wine induced significant changes on the concentration of the different salivary protein families. The lowest saliva/wine ratio (10:0.7) led to a total decrease in the concentration of P-B peptide but also significant decreases were observed for aPRPs and cystatins. However, bPRPs and gPRPs were not reduced significantly. In addition to the total decrease of P-B peptide, the saliva/wine ratio 10:2 led to a total decrease in aPRPs and statherin families. In this case, gPRPs also presented a higher decrease than for the 10:0.7 ratio, while the decrease in cystatins was quite similar to the one observed previously. For the highest saliva/wine ratio, we observed a significant decrease in almost all families of salivary proteins, except for cystatins. In this case, gPRPs were also almost totally decreased and bPRPs were significantly reduced (except for wine P1) (Figure 2). For this ratio, cystatins presented a similar decrease to the one observed for the other ratios.

These results are in agreement with previous results that indicated that P-B peptide, aPRPs, and statherin were the salivary proteins with the highest interaction towards tannins and also with other wines [22]. Furthermore, these results are also in agreement with in vivo studies that showed that these proteins were the first ones to disappear upon the interaction with a grape seed procyanidin extract [38].

Considering the wines’ effects toward the interaction with salivary proteins, for the saliva/wine ratio of 10:0.7, only aPRPs showed a different interaction. In this case, it was observed that all M wines showed a higher reduction than P wines. All the other salivary proteins presented a similar interaction with the different wines at this ratio. For the saliva/wine ratio of 10:2, gPRPs also showed a higher reduction with M wines than with P wines, while the specificity previously observed for aPRPs disappeared by a total reduction. For the saliva/wine ratio of 10:3, this discrimination between wines M and P was additionally observed for the bPRPs.

In summary, bPRPS, gPRPs, and aPRPs seem to have a different interaction with M and P wines when small or medium volumes of wine interact with each family of salivary proteins. When high wine/saliva ratios are used, these differences dissipate.

Considering the results, it was possible to discriminate between M and P wines, because the results were in agreement with the wines’ chemical characterizations. In the general characterizations, M wines presented higher total phenolic and total proanthocyanidin content, as well as higher performance on the protein precipitation assay. However, when looking at the characterization of the individual phenolic acid composition, it is interesting to observe the content of particular compounds. In M wines, caffeic acid is present at 610 μM, caftaric acid at 980 μM, and coumaric acid at 528 μM, while in P wines the concentration of these compounds is much lower 405 μM, 638 μM, and 293 μM, respectively (Table 2). These compounds have been previously linked to puckering astringency and the threshold for astringency has been reported to be 72 μM for caffeic acid, 16 μM for caftaric acid, and 139 μM for coumaric acid [39]. Since these compounds occur in much higher concentrations than their thresholds, it is expected that these phenolic acids could contribute to a more pucker sensation of M wine than P wines. In general, we observed a higher interaction with salivary proteins of M wines.

In line with what was expected from an Italian wine, the interaction with salivary proteins anticipates moderate astringency due to an average high concentration of native phenolics and tannins. In chemical terms, M wine seemed a little more evolved and could be expected to be somehow more astringent than P wine.

## Figures and Tables

**Table 1:** Red color index absorbance measurement at 520 nm and CIELab parameters obtained by MSCV coordinates program (2 mm glass path cell). Anthocyanins and carboxypyranoanthocyanins concentration obtained by High Performance Liquid Chromatography (HPLC) and express in mg/L. The presented values are the mean ± standard deviation of three independent experiments. Values with the same letter differ statistically when *p* < 0.05.

Wine	M	P
**520 nm**	0.581 ± 0.006 ^a^	0.373 ± 0.001 ^a^
**CIELab**	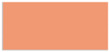	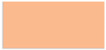
L*	69.04 ± 0.02 ^b^	78.950 ± 0.001 ^b^
a*	23.37 ± 0.03 ^c^	14.89 ± 0.05 ^c^
b*	30.20 ± 0.06 ^d^	28.4 ± 0.1 ^d^
C*	38.21 ± 0.03 ^e^	32.09 ± 0.09 ^e^
**H** ^**o**^	52.18 ± 0.09 ^f^	62.4 ± 0.1 ^f^
**[Anthocyanins] mg/L**	114 ± 3 ^g^	151 ± 4 ^g^
**[Carboxypyranoanthocyanins] mg/L**	5 ± 1 ^h^	1.4 ± 0.3 ^h^

**Table 2:** HPLC quantification of phenolic acids and their derivatives present in P and M wines. The results are expressed as mean ± standard deviation and are the result of two extractions of each wine bottle and triplicate injections. Values with the same letter differ statistically when *p* < 0.05. The *m/z* are in the negative ion mode.

Compound/[M–H]^−^ (*m/z*)	P Wines (µM)	M Wines (µM)
Gallic acid (169)	3459 ± 186 ^a^	3059 ± 46 ^a^
Methyl coumarate (177)	146 ± 32 ^b^	71 ± 5 ^b^
Methyl cinnamate (161)	129 ± 28	159 ± 7
Protocatechuic acid (153)	141 ± 15	188 ± 16
Ethyl ferulate (221)	75 ± 3 d	110 ± 2 ^d^
Caftaric acid (311)	638 ± 25 ^e^	983 ± 67 ^e^
Ethyl cinnamate (175)	242 ± 53	335 ± 9
Coutaric acid (isomer) (295)	928 ± 55 ^f^	716 ± 46 ^f^
Coutaric acid (isomer) (295)	826 ± 149 ^g^	130 ± 40 ^g^
Fertaric acid (325)	519 ± 73 ^h^	895 ± 50 ^h^
Caffeic acid (179)	405 ± 60 ^i^	611 ± 68 ^i^
Coumaric acid (163)	294 ± 31 ^j^	528 ± 21 ^j^
Syringic acid (197)	327 ± 75	278 ± 29
Ethyl cinnamate (175)	525 ± 44 ^l^	913 ± 64 ^l^
Ellagic acid (301)	173 ± 4 ^m^	330 ± 32 ^m^
Total benzoic acids	4100 ± 280	3855 ± 123
Total cinnamic acids	3610 ± 393	3863 ± 292
